# Bioinformatics Analysis of the Human Surfaceome Reveals New Targets for a Variety of Tumor Types

**DOI:** 10.1155/2016/8346198

**Published:** 2016-11-14

**Authors:** André L. Fonseca, Vandeclécio L. da Silva, Marbella M. da Fonsêca, Isabella T. J. Meira, Thayná E. da Silva, José E. Kroll, André M. Ribeiro-dos-Santos, Cléber R. Freitas, Raimundo Furtado, Jorge E. de Souza, Beatriz Stransky, Sandro J. de Souza

**Affiliations:** ^1^Brain Institute, Federal University of Rio Grande do Norte, 59064-560 Natal, RN, Brazil; ^2^Ph.D. Program in Bioinformatics, Federal University of Rio Grande do Norte, Natal, RN, Brazil; ^3^Institute of Bioinformatics and Biotechnology, Natal, RN, Brazil; ^4^Ph.D. Program in Genetics, Federal University of Para, Belém, PA, Brazil; ^5^Digital Metropolis Institute, Federal University of Rio Grande do Norte, Natal, RN, Brazil; ^6^Biomedical Engineering Department, Center of Technology, Federal University of Rio Grande do Norte, Natal, RN, Brazil

## Abstract

It is estimated that 10 to 20% of all genes in the human genome encode cell surface proteins and due to their subcellular localization these proteins represent excellent targets for cancer diagnosis and therapeutics. Therefore, a precise characterization of the surfaceome set in different types of tumor is needed. Using TCGA data from 15 different tumor types and a new method to identify cancer genes, the *S*-score, we identified several potential therapeutic targets within the surfaceome set. This allowed us to expand a previous analysis from us and provided a clear characterization of the human surfaceome in the tumor landscape. Moreover, we present evidence that a three-gene set—WNT5A, CNGA2, and IGSF9B—can be used as a signature associated with shorter survival in breast cancer patients. The data made available here will help the community to develop more efficient diagnostic and therapeutic tools for a variety of tumor types.

## 1. Introduction

Cancer genomics has gone through a dramatic period of progress due to the availability of genome-wide technologies. Large-scale projects, such as “The Cancer Genome Atlas” (TCGA, https://cancergenome.nih.gov/) and the “International Cancer Genome Consortium” (ICGC, http://icgc.org/), involve thousands of patients and have generated petabytes of data. One of the major assets of such projects is the public availability of the data allowing their integration with data from other initiatives. In that way, data from these initiatives can push a more focused and deeper analysis either in a specific gene set or in a specific cohort of patients/samples.

The human surfaceome, the collection of cell surface proteins in human cells, has been defined and studied by us previously. By using bioinformatics pipeline and an experimental approach based either on real-time PCR or on other gene expression technologies, we were able to identify potential new biomarkers for few tumor types and have characterized new cell surface putative cancer-testis (CT) antigens [1, 2]. Relevant roles of surface proteins include nutrient and ion transport, adhesion to substrates, signaling, and intercellular interaction. Due to these roles and their subcellular localization, easily accessible to therapeutic agents, surface proteins are important targets for cancer intervention. Since our original publication few reports have further explored the human surfaceome [2–6], mostly in the context of a mass-spectrometry-based characterization of the cell surface of tumor cells.

Data from TCGA/ICGC allow the development of new metrics that evaluate the frequency of gene alterations in different cancer types. Recently, we developed a new scoring system for the identification and prioritization of cancer genes [7]. The *S*-score method integrates information derived from different “omics” technologies to generate a gene-specific score that indicates whether that specific gene is a tumor suppressor (negative *S*-score) or an oncogene (positive *S*-score). The numerical value indicates the frequency in which that gene is altered in the cohort of samples used in the calculation. We have used the *S*-score metric to identify cancer genes in a set of human homologs of yeast genes characterized as suppressors of genome instability in yeast [8]. The availability of the *S*-score system provides a quantitative way to identify and prioritize cancer genes in a particular set of samples.

Here, we capitalize on the availability of data from the TCGA project to further and deeper investigate the status of the human surfaceome in 15 tumor types, including GBM and colorectal and breast tumors, all analyzed in our previous publications [1, 2]. This generated a pan-cancer landscape of the human surfaceome with the identification of shared and tumor-specific markers. Furthermore, the use of the *S*-score system allowed us to identify gene signatures associated with overall survival in breast cancer patients. These signatures can be ultimately used in the development of new and more efficient diagnostic and therapeutic protocols.

## 2. Material and Methods

### 2.1. Public Data

The protein-coding sequences of all human genes were obtained from the NCBI RefSeq (release 64) [9]. Gene ontology was retrieved from gene ontology initiative web site (http://geneontology.org/). The Illumina Human BodyMap 2.0 dataset was obtained from EMBL-EBI Expression Atlas (https://www.ebi.ac.uk/gxa/experiments/E-MTAB-513). TCGA data was retrieved from both, the TCGA web site (https://cancergenome.nih.gov/) and the cBio Cancer Genomics Portal (http://www.cbioportal.org/).

### 2.2. Identification of Transmembrane (TM) Domains in Protein-Coding Genes

To predict plasma membrane subcellular localization, the NCBI Reference Sequence dataset was submitted to TMHMM [10] version 2.0 (http://www.cbs.dtu.dk/services/TMHMM/). All sequences containing at least one TM domain were selected. To avoid false positives, sequences containing only one TM domain in the first 50 residues, which could be a signal peptide, were excluded and classified as secreted protein. Furthermore, sequences were also filtered based on the identification of signal peptide cleavage sites by SignalP, release 4.1 (http://www.cbs.dtu.dk/services/SignalP/) [11].

Since TM domains are not exclusive to cell surface proteins, the sequences were grouped according to subcellular localization as defined by gene ontology. We excluded sequences that were exclusively located at the following cellular compartments: lysosome, endoplasmic reticulum, mitochondria, cytoskeleton, endosome, liposome, nucleolus, nucleus, and ribosome. This step was conducted using in-house Perl scripts.

### 2.3. Classification of Surfaceome in GPCR, SLC, and CD

To validate the obtained list of surfaceome genes, we classified these genes as belonging to the following classes: G-protein-coupled receptors (GPCRs), solute carrier (SLC) proteins, and cluster of differentiation (CD) antigens. This was done using in-house Perl scripts. The GPCR genes were obtained from GPCRDB (http://gpcrdb.org), while CD and SLC genes were collected from HGNC (http://www.genenames.org/genefamilies/a-z#R).

### 2.4. *S*-Score Calculation for the Human Surfaceome

*S*-scores were calculated for the human surfaceome and for the 15 tumor types as previously defined [7]. The distribution of *S*-scores was used to calculate *z*-scores for all genes using R statistical package.

### 2.5. GO Enrichment Analyses

The GO enrichment analyses of the surfaceome gene cluster were conducted using clusterProfiler [12], implemented in R, with *p* values < 0.01 as a cutoff.

### 2.6. Survival

Genes with extreme *S*-score (*S*-score <−2 and >2 for breast tumors) were selected to test any putative association with overall survival in breast cancer samples derived from TCGA (without subtype distinction). Each gene was used to classify the samples into two sets, named “normal” and “altered.” The “altered” set comprised samples within which the respective genes were differentially expressed (*z*-score > 2 or *z*-score <−2, as reported by TCGA), amplified or deleted, or presenting deleterious mutations (nonsense, frameshift, and splice-site). After that, for each gene, the survival analysis was performed using the Kaplan-Meier method [13] and the difference in survival curves was tested for statistical significance using the log rank test *p* value. We then selected a nonredundant set of 20 genes with the lowest *p* value (cutoff of 0.05) and tested all possible groups of three genes.

## 3. Results and Discussion

### 3.1. A New Gene Catalog for the Human Surfaceome

**Figure 1:**
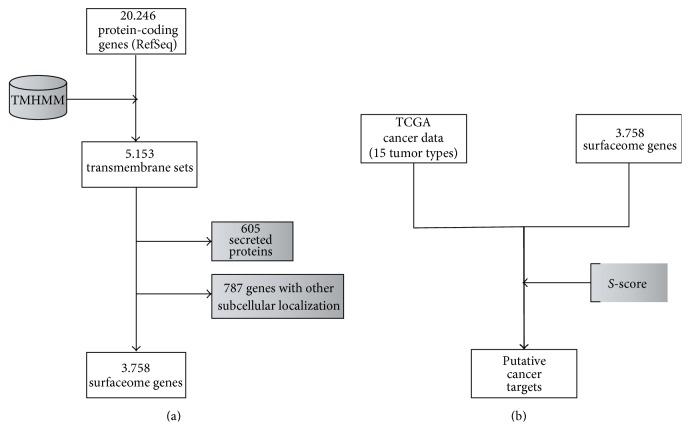
Methodology workflow. (a) The NCBI RefSeq dataset was submitted to TMHMM and selected for the presence of a transmembrane domain. Proteins containing only a signal peptide (classified as secreted) or belonging exclusively to other membranes were excluded, giving a final set of 3.758 genes coding for cell surface proteins. (b) TCGA data as used to calculate the *S*-score for the surfaceome set allowing the identification of putative surfaceome cancer genes.

First of all, we decided to reannotate the set of human genes coding for putative cell surface proteins. This was required due to (i) an improvement in the annotation of gene and protein databases regarding a protein's subcellular localization and (ii) the inclusion of new human genes in the Reference Sequence collection. The same approach used by da Cunha et al. (2009) [1] generated now a set of 3,758 human genes, composing the human surfaceome (Figure 1). The complete list of human genes coding for cell surface proteins is provided as Supplementary Table  S1, in Supplementary Material available online at http://dx.doi.org/10.1155/2016/8346198. As expected, the great majority (85%) of surfaceome genes present in our dataset in 2009 remained classified as such in 2016. New genes were added (585), mostly due to their inclusion in the Reference Sequence collection and some other genes (529) were excluded due mainly to new functional annotation that classified their protein products as belonging to other subcellular compartments.

To assess the robustness of our approach, we performed the same analysis reported by us in our original 2009 paper [1] checking the representation of three known families of cell surface proteins (G-protein-coupled receptors (GPCRs), solute carrier (SLC) proteins and cluster of differentiation (CD) antigens). Since these are large and well-studied families of cell surface proteins, we envisaged that they would be appropriate for a benchmark analysis. For GPCRs, 98% of their known members were represented in our dataset. For SLC proteins and CD antigens we found 77% and 88% represented in the surfaceome set, respectively. Overall, 90% of members of these three families were represented in our present surfaceome set, compared to 83% in our previous analysis [1]. This improvement is expected due to a better annotation of the sequences in public databases.

Capitalizing on the availability of surfaceome sets derived from mass-spectrometry analysis, we decided to compare our dataset to the dataset from Bausch-Fluck et al. [3]. For that purpose, we have only used the proteins classified as “highly confident” in [3]. Although this type of comparison is problematic for different reasons, including (i) the nonexhaustive nature of the wet-based approach (due to the method itself and the samples screened) and (ii) the different premises of both methods (the requirement of at least one TM domain per protein in our pipeline and the lack of such requirement in [3] which allowed the authors to characterize GPI-anchored proteins, e.g.), the analysis may be illuminating in the sense that it can highlight important differences in both methodologies. We found that 66.6% (664 out of 996) of the proteins classified by Bausch-Fluck et al. [3] were present in our dataset while only 17.6% (664 out of 3758) of our proteins were present in their dataset. This was expected due to the issues raised above. To illustrate the complex nature of this comparison, 23.8% of all cell surface proteins found in [3] have no TM domain, as identified by TMHMM.

### 3.2. Identification of Potential Therapeutic Targets in the Human Surfaceome

Next, the *S*-score method was used to identify cancer genes within the surfaceome set. *S*-score threshold was defined for each tumor type as the *S*-score representing the average *S*-score plus/minus three standard deviations (*z*-score ≥ 3 or ≤−3) [7]. The list of all cancer genes coding for cell surface proteins in all 15 tumors types is shown in Supplementary Table  S1. Using the above *z*-score threshold, we found 248 surfaceome genes classified as a cancer gene in at least one tumor type.

**Figure 2:**
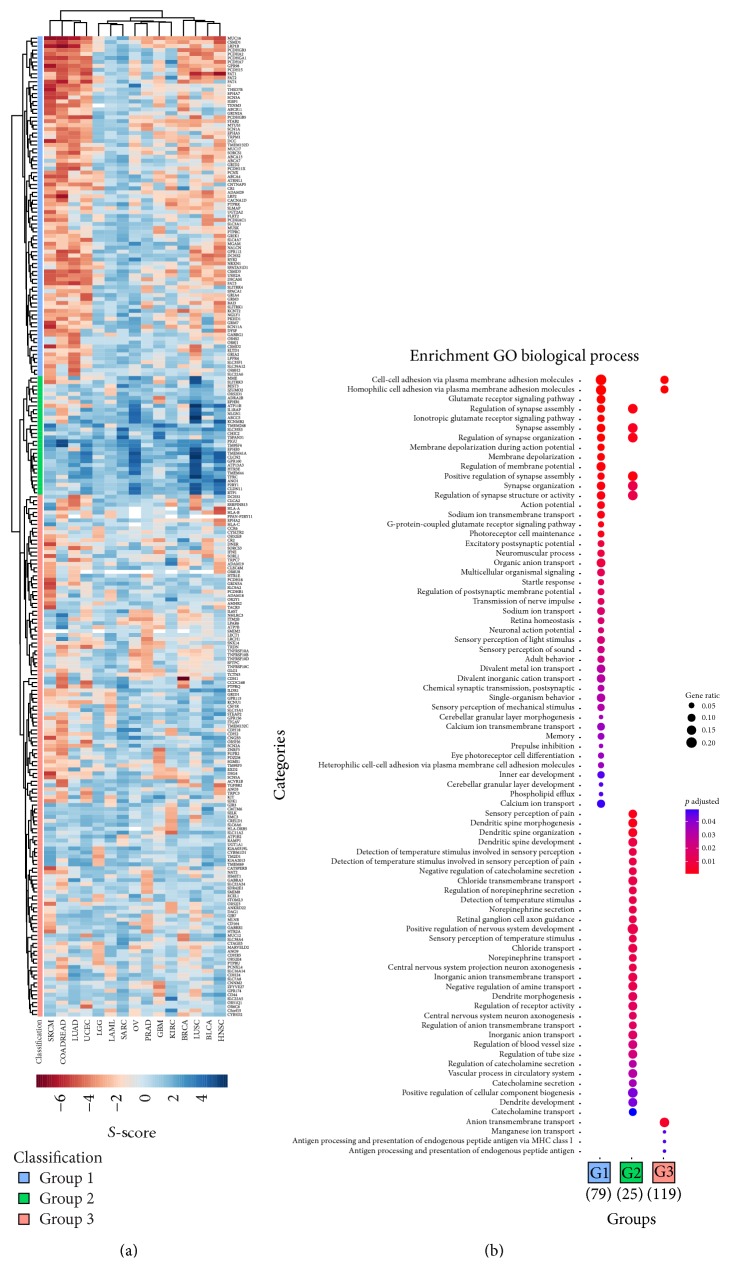
Surfaceome cancer genes identified by the *S*-score method. (a) Heatmap representation of all 248 surfaceome cancer genes for all 15 tumor types. The *S*-score distribution is represented as a range of colors (red as negative *S*-score, blue as positive *S*-scores). Tumor types are SKCM (Skin Cutaneous Melanoma), COADREAD (Colorectal Adenocarcinoma), LUAD (Lung Adenocarcinoma), UCEC (Uterine Corpus Endometrial Carcinoma), LGG (Low Grade Glioma), LAML (Acute Mieloid Leukemia), SARC (Sarcoma), OV (Ovarian Serous Carcinoma), PRAD (Prostate Adenocarcinoma), GBM (Glioblastoma), KIRC (Kidney Renal Clear Cell Carcinoma), BRCA (Breast Invasive Carcinoma), LUSC (Lung Squamous Cell Carcinoma), BLCA (Bladder Adenocarcinoma), and HSNC (Head-Neck Squamous Cell Carcinoma). (b) GO enrichment analysis for the three groups of genes as identified in (Figure 2(a)). The adjusted *p* values are sorted from least (blue) to most (red) significant. Furthermore, the dot size is based on gene ratio, which is the observed number of genes in the experimental set within the respective gene ontology category.

In the heatmap representation of the surfaceome cancer genes (Figure 2(a)) we can clearly identify three distinct clusters based on the *S*-score values for all 15 tumor types. Although all three groups have a variety of oncogenes and suppressors, some features deserve further comments. For example, the first group is mainly composed of suppressors, especially in melanoma and colorectal and lung adenocarcinoma and uterine corpus endometrial carcinoma. Genes in this group include several members of the cadherin superfamily (PCDHGB3, PCDHA2, PCDHA7, PCDH15, PCDHGB5, PCDH11X, PCDHAC1, FAT1, FAT2, and FAT4). There is a set of oncogenes in group 2 shared by almost all tumors and involving 30 genes, including EPHB1 and EPHB3. There is no clear pattern in group 3 and oncogenes and suppressors seem to be distributed evenly across all tumors.

To better understand the pattern presented in Figure 2(a), we performed a gene ontology (GO) enrichment analysis (using the “biological process” ontology) for the three different clusters. As expected, all three groups shared GO categories associated with the cell surface such as transmembrane transport and cell surface receptor signaling pathway. More interestingly, however, is the fact that specific GO categories were enriched in individual groups (Figure 2(b)). GO categories exclusively found in group 1 were clearly associated with nervous system including “neuromuscular process”; “memory”; and “neuronal action potential.” The same pattern was observed for group 2 although the GO categories represented different aspects of nervous system including the following: “sensory perception of pain” and other categories related to axonogenesis. Regarding group 3, GO analysis lent further support for the current concept of ion transport associated with cancer [14], including “manganese ion transport.” Additionally, this group presented genes related to antigen processing and presentation, highlighting that the interplay between immune and tumor cells is complex.

Several of the identified surfaceome cancer genes are known for their involvement in different aspects of cancer biology, especially the ones classified in group 2. ABCC5, a cell surface transporter, was involved in resistance to anticancer drugs [15] and overexpression of ATP11B has been linked to drug resistance in ovarian cancer [16]. Both genes were regarded as oncogenic by our work especially in lung squamous cell carcinoma and ovarian cancer. On the other hand, EPHB3 has already been suggested as a candidate target gene for both lung small cell carcinoma [17] and colorectal cancer [18]. Finally, a transferrin receptor (TFRC) has shown an increased expression in many malignant tumors [19] and was also found to be highly oncogenic in this work.

### 3.3. A Three-Gene Signature as Potential Predictor of Survival in Breast Cancer

**Figure 3:**
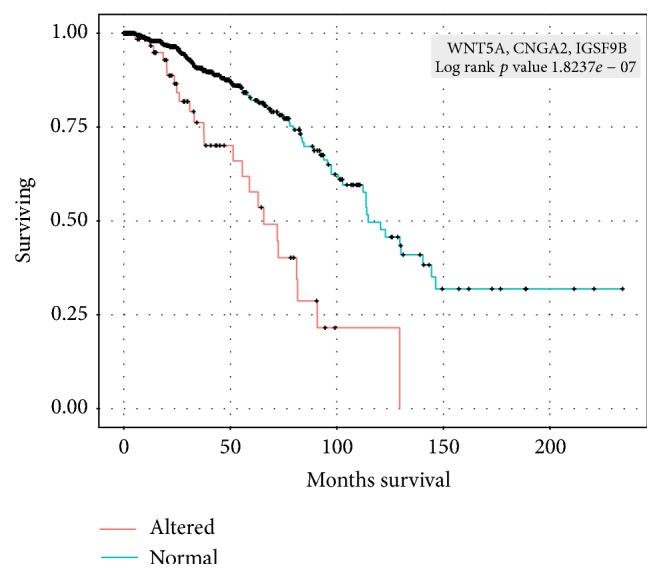
Kaplan-Meier overall survival curves in breast cancer patients. Samples were classified as having the three-gene signature (WNT5A, CNGA2, and IGSF9B) altered (red) or not (blue).

As previously discussed by us, the *S*-score method allows the prioritization of cancer genes based on clinical parameters [7]. For example, we have identified genes associated with both short- and long-term survival in ovarian cancer [7]. To test whether we could identify genes in the surfaceome set associated with clinical parameters, we decided to look at overall survival in breast tumors, since this type of tumor is the one with the largest cohort in TCGA. For this specific analysis a more relaxed threshold (*z*-score <−2 or >2) was used to classify a gene as a cancer gene in breast tumor to increase the number of genes under test without compromising the quality of the classification (a heatmap, similar to Figure 2(a) and generated using the dataset with a more relaxed threshold, is presented in Supplementary Figure  1). For each surfaceome gene classified as oncogene or suppressor in breast tumor, we split the breast cancer samples into two groups: altered (genes with differential expression, genes amplified/deleted, or genes mutated) and unaltered. For each gene, the two groups were then compared by a Kaplan-Meier analysis to evaluate whether they had significantly different overall survival. Twenty-three genes, seven oncogenes and 16 suppressors, were significantly (*p*-value < 0.05) associated with differences in overall survival in breast cancer patients (Supplementary Table  2). These genes are involved in cell adhesion and ion transport, two of the main categories enriched in our gene ontology analysis. Next, all possible combinations of these genes were similarly tested for differences in overall survival. Although we found several combinations with statistically significant differences in overall survival, we have focused on WNT5A, CNGA2, and IGSF9B due to statistical significance (it is the most significant three-gene set in Supplementary Table  2) and novelty. Patients in which one of the three genes was altered had a significantly shorter survival (*p* value = 1.82*e* ^−7^)compared to patients where these three genes were unaltered (Figure 3).

The WNT5A, CNGA2, and IGSF9B genes have negative *S*-scores in breast cancer (−2.59, −3.39, and −2.56, resp.), demonstrating a tumor suppressor profile. WNT5A belongs to the large WNT family of cysteine-rich secreted glycoproteins. The role of WNT5 in cancer is controversial. In breast cancer, the loss of WNT5A has been associated with poor prognosis [20], in agreement with the suppressor status defined by the respective *S*-score. On the other hand, WNT5A was recently reported to promote cancer cell lines invasion and proliferation [21], a feature typical of oncogenes. WNT5A is present in pathways where Wnt signaling is involved through interaction with Frizzleds (FZD10, e.g.) and Dishevelled. WNT5A tumor suppressor profile change Wnt signaling characteristic leading to cancer [22]. CNGA2, a homotetrameric channel in olfactory sensory neurons [23], has not been reported in association with cancer. However, CNGA2 represents the alpha subunit of a cyclic nucleotide-gated olfactory channel possessing a role in calcium signaling pathway acting through calmodulin-like 6 and calcium/calmodulin-dependent protein kinase IV [24] directly involved with protein kinase A (PKA), a biological target in cancer therapy. IGSF9B was only recently identified as an inhibitory synaptic adhesion molecule [25] and no link with cancer was found in the literature.

## 4. Conclusion

We have updated the set of human genes coding for cell surface proteins, the human surfaceome. Using TCGA data for 15 tumor types and a new method of cancer genes classification that integrates information from different “omics” technologies and allows a ranking based on clinical parameters (*S*-score), we identified several potential therapeutic targets within the surfaceome set. Furthermore, we present evidence that a three-gene set—WNT5A, CNGA2 and IGSF9B—was associated with shorter survival in breast cancer patients. Our results clearly show the importance of large-scale genomics datasets from cancer patient cohorts, like the one provided by TCGA. We envisage that the data we provide here will be extremely useful to researchers who aim to characterize cell surface targets for a variety of tumor types.

## Supplementary Material

The supplementary material contains: (i) a list of all surfaceome gene with the corresponding S-score for all tumors used in this report (Supplementary Table 1); (ii) a list of three gene sets tested for differences in overall survival (Supplementary Table 2) and (iii) a heatmap, similar to Figure 2A, showing surfaceome genes identified using a more relaxed threshold.

## References

[1] da Cunha J. P. C., Galante P. A. F., de Souza J. E. (2009). Bioinformatics construction of the human cell surfaceome. *Proceedings of the National Academy of Sciences of the United States of America*.

[2] Da Cunha J. P. C., Galante P. A. F., De Souza J. E. S. (2013). The human cell surfaceome of breast tumors. *BioMed Research International*.

[3] Bausch-Fluck D., Hofmann A., Bock T. (2015). A mass spectrometric-derived cell surface protein atlas. *PLoS ONE*.

[4] Town J., Pais H., Harrison S. (2016). Exploring the surfaceome of Ewing sarcoma identifies a new and unique therapeutic target. *Proceedings of the National Academy of Sciences of the United States of America*.

[5] Hofmann A., Thiesler T., Gerrits B. (2015). Surfaceome of classical Hodgkin and non-Hodgkin lymphoma. *Proteomics—Clinical Applications*.

[6] de Souza J. E. S., Galante P. A. F., de Almeida R. V. B. (2012). SurfaceomeDB: a cancer-orientated database for genes encoding cell surface proteins. *Cancer Immunity*.

[7] de Souza J. E. S., Fonseca A. F., Valieris R. (2014). S-score: a scoring system for the identification and prioritization of predicted cancer genes. *PLoS ONE*.

[8] Putnam C. D., Srivatsan A., Nene R. V. (2016). A genetic network that suppresses genome rearrangements in Saccharomyces cerevisiae and contains defects in cancers. *Nature Communications*.

[9] Pruitt K. D., Tatusova T., Brown G. R., Maglott D. R. (2012). NCBI Reference Sequences (RefSeq): current status, new features and genome annotation policy. *Nucleic Acids Research*.

[10] Krogh A., Larsson B., Von Heijne G., Sonnhammer E. L. L. (2001). Predicting transmembrane protein topology with a hidden Markov model: application to complete genomes. *Journal of Molecular Biology*.

[11] Petersen T. N., Brunak S., von Heijne G., Nielsen H. (2011). SignalP 4.0: discriminating signal peptides from transmembrane regions. *Nature Methods*.

[12] Yu G., Wang L.-G., Han Y., He Q.-Y. (2012). clusterProfiler: an R package for comparing biological themes among gene clusters. *OMICS*.

[13] Kaplan E. L., Meier P. (1958). Nonparametric estimation from incomplete observations. *Journal of the American Statistical Association*.

[14] Beroukhim R., Getz G., Nghiemphu L. (2007). Assessing the significance of chromosomal aberrations in cancer: methodology and application to glioma. *Proceedings of the National Academy of Sciences of the United States of America*.

[15] Borst P., Evers R., Kool M., Wijnholds J. (2000). A family of drug transporters: the multidrug resistance-associated proteins. *Journal of the National Cancer Institute*.

[16] Moreno-Smith M., Halder J. B., Meltzer P. S. (2013). ATP11B mediates platinum resistance in ovarian cancer. *The Journal of Clinical Investigation*.

[17] Kang J. U., Koo S. H., Kwon K. C., Park J. W., Kim J. M. (2009). Identification of novel candidate target genes, including *EPHB3*, *MASP1* and *SST* at 3q26.2-q29 in squamous cell carcinoma of the lung. *BMC Cancer*.

[18] Cortina C., Palomo-Ponce S., Iglesias M. (2007). EphB-ephrin-B interactions suppress colorectal cancer progression by compartmentalizing tumor cells. *Nature Genetics*.

[19] Ryschich E., Huszty G., Knaebel H. P., Hartel M., Büchler M. W., Schmidt J. (2004). Transferrin receptor is a marker of malignant phenotype in human pancreatic cancer and in neuroendocrine carcinoma of the pancreas. *European Journal of Cancer*.

[20] Dejmek J., Dejmek A., Säfholm A., Sjölander A., Andersson T. (2005). Wnt-5a protein expression in primary Dukes B colon cancers identifies a subgroup of patients with good prognosis. *Cancer Research*.

[21] Shojima K., Sato A., Hanaki H. (2015). Wnt5a promotes cancer cell invasion and proliferation by receptor-mediated endocytosis-dependent and -independent mechanisms, respectively. *Scientific Reports*.

[22] Klaus A., Birchmeier W. (2008). Wnt signalling and its impact on development and cancer. *Nature Reviews Cancer*.

[23] Nache V., Eick T., Schulz E., Schmauder R., Benndorf K. (2013). Hysteresis of ligand binding in CNGA2 ion channels. *Nature Communications*.

[24] Trudeau M. C., Zagotta W. N. (2003). Calcium/calmodulin modulation of olfactory and rod cyclic nucleotide-gated ion channels. *Journal of Biological Chemistry*.

[25] Woo J., Kwon S.-K., Nam J. (2013). The adhesion protein IgSF9b is coupled to neuroligin 2 via S-SCAM to promote inhibitory synapse development. *Journal of Cell Biology*.

