# Supplementary material for: Bioinformatics Analysis of the Human Surfaceome Reveals New Targets for a Variety of Tumor Types
Source: Int J Genomics. 2016 Nov 14;2016:8346198. doi: 10.1155/2016/8346198 (PMC5206789; doi:10.1155/2016/8346198)
Supplement: Supplementary file 3 [file 8346198.f3.pdf]

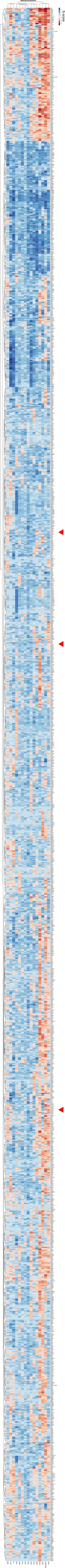
Heatmap Surfaceome
S-score
4
2
0
−2
−4
−6
